# Circulating tumor DNA in cholangiocarcinoma: current clinical applications and future perspectives

**DOI:** 10.3389/fcell.2025.1616064

**Published:** 2025-07-02

**Authors:** Yi Wang, Yike Li, Zhong Liang, Yuqiao Zhang, Tong Li, Chenjun Tian, Jinyu Zhao, Boru Jin, Jie Cao, Yanyan Lin

**Affiliations:** ^1^ The First School of Clinical Medicine, Lanzhou University, Lanzhou, China; ^2^ Department of Urology, The First People’s Hospital of Lanzhou City, Lanzhou, China; ^3^ West China School of Medicine, Sichuan University, Chengdu, China; ^4^ Department of laboratory, The First Hospital of Lanzhou University, Lanzhou, China; ^5^ Department of General Surgery, The First Hospital of Lanzhou University, Lanzhou, China

**Keywords:** cholangiocarcinoma, circulating tumor DNA, liquid biopsy, prognosis monitoring, tumor-informed ctDNA

## Abstract

Cholangiocarcinoma is a highly heterogeneous malignant tumor, including intrahepatic cholangiocarcinoma, hepatoportal cholangiocarcinoma and distal cholangiocarcinoma. Its incidence is increasing worldwide and currently accounts for approximately 15% of all primary liver cancers and 3% of all gastrointestinal malignancies. There is a lack of early diagnostic methods for cholangiocarcinoma, and the overall treatment effect is poor, with a 5-year survival rate of less than 25%. New biomarkers are urgently needed in clinical practice to improve the current diagnosis and treatment status. Circulating tumor DNA (ctDNA) is DNA fragments released by tumor cells, which can show tumor-specific gene mutations (such as IDH1/2, FGFR2 fusion) and epigenetic modifications (such as abnormal methylation). With the rapid development of tumor liquid biopsy technology, ctDNA has been gradually applied in solid tumors such as lung cancer and colorectal cancer due to its high sensitivity and dynamic monitoring capabilities. This review systematically introduces ctDNA technology and its progress in early screening, early diagnosis, treatment response, and prognosis monitoring of cholangiocarcinoma. In addition, this review also summarizes the challenges and limitations of current ctDNA technology and analyzes future hot research directions.

## 1 Background

Cholangiocarcinoma (CCA) is a highly heterogeneous malignant tumor originating from the bile duct epithelium, and its pathological type is mostly adenocarcinoma. CCA can be divided into intrahepatic cholangiocarcinoma (iCCA) and extrahepatic cholangiocarcinoma (eCCA) according to the anatomical location. ECCA includes hilar cholangiocarcinoma (hCCA) and distal cholangiocarcinoma (dCCA). There are significant differences in the treatment strategies and prognosis of CCA in different anatomical locations (Valle et al., 2021). In recent years, the incidence of CCA has continued to increase, accounting for about 3% of gastrointestinal malignancies. Among them, iCCA is the second most common primary liver cancer after hepatocellular carcinoma, accounting for about 15% (Banales et al., 2020). There are significant regional differences in the incidence of CCA, with Asia having the highest incidence of CCA worldwide. The age-standardized incidence is highest in northeastern Thailand (85 cases/100,000), followed by Gwangju, South Korea (8.8 cases/100,000). The incidence in the West is lower than in Asia, with the highest incidence in Italy (3.4 cases/100,000) (Khan et al., 2019; Qurashi et al., 2025). The number of biliary tract cancer (BTC) cases worldwide has increased by 84.8% in the past 20 years (1990–2019), and the number of new cases in China has increased by 211% (Chen et al., 2022; Su et al., 2024). As the population ages, the incidence of BTC in China is expected to continue to rise in the next decade. In addition, CCA is highly malignant and lacks specific early detection methods. Most patients are already in the late stage when diagnosed, missing the opportunity for surgical treatment (Moss et al., 2007). The prognosis of CCA is poor, with a 5-year overall survival rate (OS) of less than 25% (Kamsa-Ard et al., 2020).

Tumor liquid biopsy, as an emerging minimally invasive sampling and detection method, focuses on detecting blood or body secretions, such as tumor cells, molecules and metabolites. Compared with traditional tissue biopsy, liquid biopsy has the advantages of simple operation and minimal invasiveness, which significantly improves patient acceptance and examination feasibility (Ma et al., 2024). As emerging liquid biopsy biomarkers, cell free DNA (cfDNA) and circulating tumor DNA (ctDNA) have shown great potential in cancer treatment and monitoring. ctDNA is a tumor-derived DNA fragment released from tumor cells through apoptosis, necrosis or active secretion, which can reflect the genomic and epigenomic characteristics of the tumor (Diehl et al., 2008). The dynamic changes of its concentration have been proven to be closely related to tumor burden and microenvironment characteristics, and it is more suitable for tumor efficacy evaluation than protein markers such as CA19-9 (Heitzer et al., 2019). With the advancement of technologies such as real-time quantitative PCR (rt-qPCR), digital droplet PCR (ddPCR), Sanger sequencing, and next-generation sequencing (NGS), it is expected that the detection of gene mutations and abnormal DNA methylation in ctDNA will replace tumor pathological biopsy and tumor marker detection (Ståhlberg et al., 2017; Hannigan et al., 2019; Mody et al., 2019; Olmedillas-López et al., 2022).

Currently, the application of ctDNA in CCA is mostly limited to small sample exploratory studies and lacks standardized testing procedures. This review will systematically review the scientific research evidence on the early diagnosis, efficacy response and prognosis detection of CCA for the first time, and explore the prospects and research hotspots of ctDNA in the clinical diagnosis and treatment of CCA.

## 2 The biological origin, molecular characteristics and detection methods of ctDNA

**FIGURE 1:**
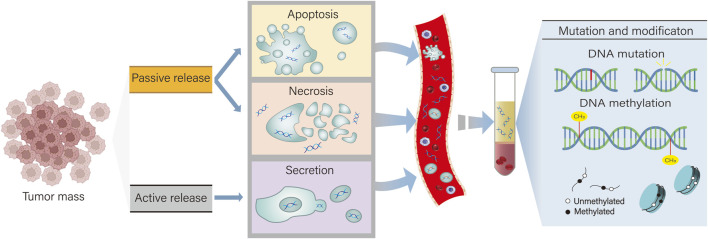
ctDNA has two types of release: active secretion and passive release. Active secretion refers to the secretion of ctDNA by tumor cells via exosomes or microvesicles. Passive release of ctDNA occurs when cells undergo apoptosis and necrosis. By detecting DNA mutations and abnormal methylation in ctDNA, it can assist in the diagnosis and treatment of cholangiocarcinoma.

ctDNA is a circulating free DNA (cfDNA) that is released into biological fluids by cancer cells through apoptosis, necrosis, or active release (Normanno et al., 2025). cfDNA in normal humans is mainly derived from white blood cells and stromal cells and is rapidly cleared within a few minutes to 1–2 h (Sun et al., 2015; Li et al., 2023). On the one hand, compared with cfDNA, the base fragments of ctDNA from cancer patients are about 20–50 base pairs, which is significantly shorter than cfDNA from normal cells (Underhill et al., 2016). This feature makes it more stable than cfDNA, and ctDNA can still provide reliable monitoring data when tumor heterogeneity is large. On the other hand, ctDNA has a short half-life (about 15 min) and can be used as a real-time tumor marker to quickly reflect the dynamic changes of the tumor (Ma et al., 2024). It is these two characteristics of ctDNA that give it obvious advantages over traditional biopsy markers (Figure 1 Mechanism of ctDNA release).

Currently, ctDNA detection methods mainly include mutation detection based on digital PCR (dPCR), next-generation sequencing (NGS), and whole genome sequencing (WGS), etc. These methods have their own advantages and disadvantages, but all have the potential to be used for early monitoring of tumors, real-time detection of dynamic changes, and monitoring of disease progression (Table 1 Comparison of methods for ctDNA detection).

**TABLE 1:** Comparison of methods for ctDNA detection.

Technology type	Technology Name	Core Principle	Sensitivity	Advantages	Disadvantages	Clinical applications
PCR Technology	Digital PCR (dPCR) (Hindson et al., 2011; ZHANG et al., 2015; Zhang et al., 2022)	Single-molecule absolute quantification through droplet partitioning	0.01%–0.10%	High sensitivity Low cost	Only detects known mutations	Detection of specific point mutations, copy number variations, insertions, deletions, and gene fusions
	BEAMing Technology (García-Foncillas et al., 2017; Li et al., 2019)	Combination of emulsion PCR and flow cytometry	0.01%	Same as dPCR	Same as dPCR	Early screening and low-abundance detection
Targeted Sequencing Technology	Tagged Amplicon Deep Sequencing (TAm-Seq) (Forshew et al., 2012; Nikanjam et al., 2022)	Targeted amplicon enrichment and high-throughput sequencing	>2%	High sensitivity Can detect both known and novel mutations	High cost	Dynamic monitoring of mutation frequency changes
	Cancer Personalized Profiling by deep sequencing (CAPP-Seq) (Nikanjam et al., 2022)	Probe capture of preset cancer-related genes combined with deep sequencing	0.01%	Same as TAm-Seq	Same as TAm-Seq	Monitoring treatment efficacy and tracking acquired resistance mutations
Whole Genome Sequencing Technology	Whole Genome Sequencing (WGS) (Wan et al., 2017; McGuire et al., 2008; Imperial et al., 2019)		>1%	Can detect novel mutations Whole genome coverage	Low sensitivity High cost	Comprehensive evaluation and analysis of tumor genome genetic characteristics
	Whole Exome Sequencing (WES) (Imperial et al., 2019; Li et al., 2019)		5%–10%	Can detect novel mutations Whole exome coverage	Low sensitivity High cost	Identifying potential oncogenes and tumor suppressor genes in coding regions
Epigenetic Analysis Technology	Whole Genome Bisulfite Sequencing (WGBS) (Hon et al., 2012; Wardenaar et al., 2013; Wasenang et al., 2019; Liu et al., 2020)	Bisulfite treatment followed by genome-wide methylation site detection		Most comprehensive and information-rich DNA methylome analysis	High cost Technically complex	Analysis of methylation levels in specific domains of cancer cells for early tumor diagnosis

In patients who obtain tissue specimens, the combined use of multiple detection methods to perform tumor-informed ctDNA testing can further improve the ctDNA detection rate. Tumor-informed ctDNA testing refers to the development of a custom assay panel based on the patient’s tumor tissue sequencing results (such as WES/WGS) to perform more in-depth sequencing of known mutations in the tumor. Research results in non-small cell lung cancer, pancreatic cancer, colorectal cancer, and breast cancer show that tumor-informed ctDNA detection technology can significantly increase the chances of capturing and identifying ctDNA fragments carrying targeted mutations, achieving high-sensitivity detection at low ctDNA concentration levels, and is particularly suitable for minimal residual disease (MRD) monitoring and recurrence warning (Watanabe et al., 2022; Chen et al., 2023; Santonja et al., 2023; Nesic et al., 2024; Sasaki et al., 2025). Currently, there are few reports on this technology in CCA.

Tumor-naive ctDNA testing does not rely on tissue specimens and is used for preliminary screening or in scenarios where tissue is unavailable. It has the advantages of being non-invasive, capable of dynamic monitoring, and having a wide detection range. The results of the study showed that tumor-naive ctDNA testing could assist in the early diagnosis of CCA and guide custom treatment selection (such as initial screening of targeted drugs), but the sensitivity depends on the ctDNA concentration, and negative results are more likely to occur when the tumor burden is low (Miura et al., 2024).

## 3 Characteristics of ctDNA in CCA

In CCA, due to severe tumor interstitial fibrosis and sparse vascular distribution, the efficiency of ctDNA release is significantly lower than that of other solid tumors (such as colorectal cancer). However, its fragment characteristics (such as short fragmentation, and terminal oxidative damage) and epigenetic abnormalities are still highly heterogeneous (Nakamura et al., 2015; Cristiano et al., 2019). The results of ctDNA testing are highly consistent with those of tissue biopsies, with a sensitivity of up to 84.8% in terminal cancer patients (Hwang et al., 2024). Through non-invasive liquid biopsy technology, ctDNA can comprehensively reflect the genomic characteristics of CCA, including high-frequency driver gene mutations, signal pathway abnormalities, and pathological classification information, providing a basis for personalized treatment.

### 3.1 Common ctDNA mutations and their clinical significance

#### 3.1.1 KRAS

KRAS mutations were more frequently detected in eCCA, with G12D (37.0%), G12V (24.0%), and Q61H (8.2%) being the main sites. KRAS G12/13 mutation is an important prognostic marker for CCA and is closely associated with shortened OS and recurrence-free survival (RFS) (sensitivity 80%, specificity 93%). Meanwhile, ddPCR analysis of plasma ctDNA showed that patients with higher KRAS G12/G13 mutant allele frequency (MAF) and CA19-9 levels (MAF >0.174% and CA19-9 >49.99 U/mL) had a significantly worse prognosis (15.8 vs. 39.0 months; p = 0.046) (Thongyoo et al., 2025). It can be found that ctDNA detection found that KRAS is associated with poor survival in CCA. This approach offers a less invasive diagnostic alternative to traditional biopsy methods and provides critical insights into the potential of cfDNA analysis to act as a predictive tool for patient survival. Although targeted therapies for the KRAS G12C mutation, such as sotolacizumab, have been approved by the FDA, the development of inhibitors targeting other KRAS mutation subtypes is still in its early stages. In addition, studies have shown that inhibition of casein kinase 2 (CK2) may affect metabolic pathways in KRAS mutant CCA, providing a new research direction for targeting KRAS-driven tumor metabolism and is expected to provide a potential new approach for the treatment of CCA (Lee D. S. et al., 2024). In addition, KRAS mutations induce high expression of PD-L1/CTLA-4 by activating the MAPK pathway, forming an immunosuppressive microenvironment, thereby leading to resistance to immunotherapy (Job et al., 2020).

#### 3.1.2 TP53

Homologous recombination repair (HR) pathway gene mutations (such as TP53, BRCA2 and RAD51D) have also been shown to be associated with a poor prognosis in CCA. TP53 mutation is the most common mutation type in CCA, with a detection rate of up to 38.1% (Silverman et al., 2019). Sanger sequencing of bile cfDNA revealed that patients with HR pathway mutations had significantly shorter survival than those without mutations (P = 0.0049), which may be related to the accelerated tumor progression caused by genomic instability caused by DNA damage repair defects (Yin et al., 2024). In addition, the study also found that patients with HR pathway mutations may be more sensitive to PARP inhibitors (such as olaparib), suggesting that it can be used as a biomarker for targeted therapy to improve patient prognosis. However, there are still few clinical trials of PARP inhibitors for CCA, and their efficacy needs to be further verified.

#### 3.1.3 IDH1

It has been reported that approximately 13%–25% of iCCA patients have isocitrate dehydrogenase 1 (IDH1) mutations (Rizzo et al., 2021). Berchuck et al. performed NGS analysis on ctDNA in the blood of 1,671 patients with advanced BTC, and the results showed that 9.1% of patients had IDH1 mutations. Among patients with IDH1 mutations detected in tissues, 87% (41/47) of patients also had these mutations detected in ctDNA. This shows that IDH1 mutations have high tissue consistency and can be used for targeted therapy (Berchuck et al., 2022). Ivosidenib, an oral, selective mutant IDH1 inhibitor, has shown promising results in a Phase III trial, significantly improving progression-free survival (PFS) and OS in advanced CCA (Abou-Alfa et al., 2020a). Resistance is particularly evident in IDH1-targeted therapies and may be related to acquired resistance mutations in IDH1 or IDH2 that prevent ivosidenib from binding to its target site (Cleary et al., 2022).

#### 3.1.4 FGFR

The incidence of rearrangement or fusion changes (3.5%) was higher than that of amplification (2.6%), and mutation events were rare (0.9%) (Helsten et al., 2016). Alberto et al. conducted a retrospective study of 18 iCCA patients who were found to have FGFR2 fusions. Plasma samples were analyzed using a custom hybrid capture gene panel with NGS (VHIO-iCCA panel). The results showed that patients in the high ctDNA group had a worse prognosis than those in the low ctDNA group (median PFS 6.53 months vs. 13.3 months, P = 0.0018; median OS 10.6 months vs. 21.2 months, P = 0.0198). In addition, 16 patients (88.9%) were found to have positive FGFR2 fusion events in plasma ctDNA, proving that VHIO-iCCA can accurately detect FGFR2 fusion in plasma ctDNA, thereby quickly screening patients who benefit better from targeted therapy. (González-Medina et al., 2024). A variety of FGFR receptor inhibitors have been developed, such as infigratinib, futibatinib, pemigatinib, erdafitinib and derazantinib, etc., and phase I and phase II trials of these drugs have shown good results (Javle et al., 2018; Goyal et al., 2019; Abou-Alfa et al., 2020b; Bahleda et al., 2019; Mazzaferro et al., 2019). Goyal et al. reported evidence of acquired resistance to FGFR inhibitors in CCA. PCR and Sanger sequencing of plasma ctDNA were performed in three patients receiving FGFR inhibitors, and the results showed that the gatekeeper mutation FGFR2 V564F was found in all patients, suggesting that secondary mutations in the FGFR2 kinase domain are a potential resistance mechanism (Goyal et al., 2017).

## 4 Clinical application of ctDNA in CCA

**FIGURE 2:**
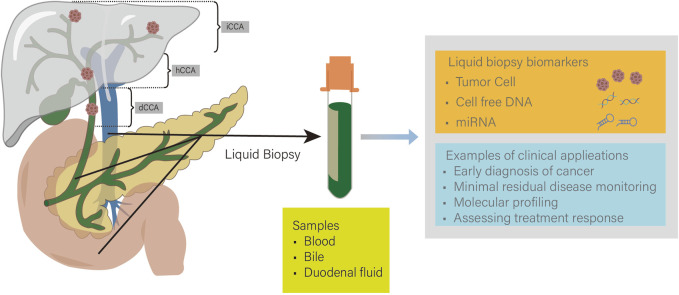
iCCA, intrahepatic cholangiocarcinoma; hCCA, hilar cholangiocarcinoma; dCCA, distal cholangiocarcinoma. Tumor liquid biopsy techniques are widely used in cholangiocarcinoma (CCA). Common samples include blood, bile, and duodenal fluid. Detection of tumor cells, cell free DNA (cfDNA), and microRNA (miRNA) can enable early diagnosis and personalized treatment of CCA.

CCA is a malignant tumor originating from the bile duct epithelium. Due to its lack of specific clinical symptoms, most patients are diagnosed at an advanced stage (Moss et al., 2007). Advanced CCA has poor treatment efficacy, so early diagnosis can significantly reduce the mortality of CCA and is crucial to improving patient prognosis. In recent years, with the continuous development of biomarkers and detection technologies, early diagnosis methods for CCA have continued to improve (Figure 2 Tumor liquid biopsy for CCA).

### 4.1 Early diagnosis and screening of CCA

#### 4.1.1 Traditional diagnostic methods

Imaging examinations (ultrasound/CT/MRI) are still the first-line tools for CCA screening. Common manifestations include bile duct obstruction, dilatation, and masses. They can evaluate tumor morphology (such as local invasion, vascular encapsulation) and metastasis status (Khan et al., 2012; Rushbrook et al., 2024). However, imaging methods have low sensitivity for small tumors and early lesions, and can only provide structural information of the tumor, but cannot reveal the molecular biological characteristics of the tumor and pathological changes at the cellular level (European Association for the Study of the Liver, 2023). Endoscopic examination techniques such as endoscopic ultrasound (EUS), magnetic resonance cholangiopancreatography (MRCP) and endoscopic retrograde cholangiopancreatography (ERCP) can directly observe bile duct lesions, detect early lesions and tiny tumors, and can obtain pathological information when combined with tissue biopsy. For example, ERCP combined with brush cytology can obtain tissue or cells, but its sensitivity is low (45%), so negative cytology does not exclude the diagnosis of CCA (Navaneethan et al., 2015). However, endoscopic examination is greatly affected by the quality of tissue samples and the sampling location, and has low sensitivity. In addition, the entry of the endoscope into the bile duct may lead to related complications, such as pancreatitis and bleeding (Tamada et al., 2011). Pathological biopsy is still the gold standard for diagnosing bile duct cancer. Pathological diagnosis can be performed directly through brush cytology, fine needle puncture or percutaneous methods. However, tissue specimens are difficult to obtain and can only reflect information about the sampling site, which is greatly affected by sample heterogeneity. Moreover, as an invasive examination, it is easy to cause harm to patients and is not convenient for continuous monitoring of disease progression (Vaidyanathan et al., 2018).

Compared with the above traditional methods, liquid biopsy is a minimally invasive sample collection method, especially suitable for patients or high-risk groups who cannot obtain tissue samples (Heitzer et al., 2019). Carbohydrate antigen 19–9 (CA19-9) is currently the most commonly used serum tumor marker for CCA diagnosis. Its false positive rate in patients with biliary obstruction exceeds 50%, and Lewis antigen-negative individuals (about 10% of the population) cannot secrete CA19-9, which seriously limits its universality (Ballehaninna and Chamberlain, 2012; Liang et al., 2015; Luo et al., 2016). The American Association for the Study of Liver Diseases and the American College of Gastroenterology both pointed out that the clinical application of CA19-9 faces limitations in CCA (European Association for the Study of the Liver, 2022; Boberg et al., 2006; Levy et al., 2005; Sinakos et al., 2011; Lindor et al., 2015; Bowlus et al., 2023).

#### 4.1.2 The potential of ctDNA as a non-invasive early diagnostic tool

In recent years, ctDNA has demonstrated significant advantages in the early diagnosis, disease monitoring and prognosis assessment of CCA due to its non-invasive, real-time monitoring, and dynamic change monitoring. Genetic and epigenetic changes in ctDNA are closely related to changes in tumor tissue, which lay the foundation for the application of ctDNA in CCA (Ettrich et al., 2019). Currently, cfDNA detection of CCA has been achieved through multiple sample sources such as blood, bile, and duodenal fluid (Table 2 Comparison of liquid biopsy specimens for CCA).

**TABLE 2:** Tumor liquid biopsy of CCA.

Sample	Sampling Method	Advantages	Disadvantages	Clinical Applications
Blood	Venous blood draw	Easy to obtain	Low sensitivity in early stages	Early screening and monitoring of high-risk populations evaluation of treatment response
Bile	ERCP, PTCD	High sensitivity	Difficult to collect	Detection of targetable mutations guiding treatment plans
Duodenal Fluid	Gastric tube, ERCP	High sensitivity	Difficult to collect	Combined detection of biliary and pancreatic system tumors

Blood ctDNA: Multiple studies have confirmed that blood ctDNA is as effective as traditional tissue genomic analysis and can be used as a marker for the diagnosis of CCA (Wasenang et al., 2019; Yang J. D. et al., 2021; Awosika et al., 2024). Hwang S et al. showed that the ctDNA in the blood of patients with BTC was consistent with the gene profile of tumor tissue, with a sensitivity of 84.8% and a positive predictive value (PPV) of 79.4% (Hwang et al., 2024).

Bile cfDNA: As the environment for the growth of bile duct tumor cells, bile is rich in tumor markers released by tumor cells through paracrine or autocrine pathways, making the detection of bile tumor markers an important means for early diagnosis of bile duct cancer and monitoring of disease progression (Shu et al., 2024; Liu et al., 2023). Multiple studies have reported that the mutation spectrum of bile cfDNA (such as TP53, KRAS, IDH1) is highly consistent with that in tumor tissue (P < 0.001) (Shen et al., 2019; Arechederra et al., 2022; Yin et al., 2024). By combining machine learning to optimize variant screening methods, the sensitivity of bile cfDNA detection can be increased by 2 times (Ito et al., 2024). Moreover, the content of bile cfDNA is much higher than that of plasma ctDNA, reaching 68.2 times that of plasma. The detection rate of driver mutations (bile 54% vs. plasma 17%) and sensitivity (96.2% vs. 31.6%) are significantly higher than those in plasma (Driescher et al., 2020; Arrichiello et al., 2022; Ito et al., 2024; Yin et al., 2024). Although bile sample acquisition is more technically demanding than blood sampling, biliary drainage constitutes an integral part of the initial therapeutic protocol for CCA patients with obstructive jaundice. Consequently, bile cfDNA analysis is particularly suitable for patients with biliary tract obstruction (Kearney et al., 2023; Shu et al., 2024).

Duodenal fluid ctDNA: Duodenal fluid (DF) contains components from the bile duct, pancreatic duct, gastric juice, and intestinal juice, and is mainly affected by pancreatic juice and bile. A study on DF analysis found that the cfDNA concentration of DF was significantly higher than that of plasma cfDNA, and it was more advantageous than plasma cfDNA in detecting low-abundance variants, and could reflect the overall microenvironment of the pancreatic and biliary system (González-Medina et al., 2024; Tavano et al., 2024). Compared with the precise detection of CCA using bile cfDNA, DF is more suitable for the combined detection of biliary and pancreatic system tumors.

In addition to detecting specific mutations in ctDNA, cancer type-specific methyl groups can also be found (Khosla et al., 2024). Wasenang et al. used a qPCR-based methylation-sensitive high-resolution melting (MS-HRM) method and found that hypermethylation of OPCML, HOXA9, and HOXD9 genes in serum ctDNA can effectively distinguish CCA from benign biliary diseases (such as gallstones), with a sensitivity and specificity of 62.5% and 100%, respectively (Wasenang et al., 2019). The NGS-based targeted methylation detection system developed by the Circulating Cell-free Genome Atlas (CCGA; NCT02889978) Consortium has validated the diagnostic value of cfDNA methylation patterns in a multi-cancer cohort study covering CCA (Liu et al., 2020; 2018). This technology can simultaneously analyze hundreds of methylation sites, significantly improving the detection throughput. Yang et al. found that the sensitivity of reduced-representation bisulfite sequencing (RRBS) technology in the early detection of ctDNA methylation combination markers can reach 76% and the specificity is 94% (Yang J. D. et al., 2021). Moreover, among patients with low levels of the traditional marker serum CA19-9 (≤100 U/mL), the test was able to successfully identify 64% of patients who were suitable for transplantation or surgical resection. The above research results show that ctDNA methylation markers can be effectively used as an early screening and identification tool for CCA. In the future, its clinical application can be further broadened through combined detection with other tumor biopsy markers.

In summary, ctDNA and bile cfDNA have great clinical potential for the accurate detection of CCA-related gene mutations. They can not only make up for the shortcomings of tissue biopsy, but provide a more sensitive and accurate detection method for early diagnosis, disease progression monitoring and targeted therapy of CCA.

In the future, the integration of liquid biopsy with imaging genomics and artificial intelligence is expected to achieve full-cycle management of CCA: “Non-invasive early-stage cancer detection, precise classification, and dynamic monitoring”.

### 4.2 Clinical prognostic assessment of CCA

#### 4.2.1 Current clinical prognosis of CCA

Early-stage CCA lacks specific clinical and diagnostic methods and has a poor prognosis, with a 5-year OS rate of only 3%–24% (Tawarungruang et al., 2021). After receiving the standard treatment of surgery combined with adjuvant therapy, 60%–70% of patients will still experience recurrence, and the 5-year survival rate is usually less than 25% (Brandi et al., 2012; Banales et al., 2016; Blechacz, 2017). Monitoring tools related to CCA prognosis are relatively lacking, and expanding prognostic monitoring methods is crucial to improving the clinical management of CCA.

#### 4.2.2 Clinical value of ctDNA prognostic assessment

Although CA19-9 and carcinoembryonic antigen (CEA) are widely used in the diagnosis and disease monitoring of CCA in clinical practice, their sensitivity and specificity are still low, and their role as independent prognostic markers is limited. In recent years, ctDNA has shown potential as a powerful prognostic biomarker for postoperative MRD in multiple cancer types (Huffman et al., 2022; Kotani et al., 2023; Hou et al., 2022). ctDNA can be used to detect tiny residual lesions early after surgery, thereby providing more accurate disease monitoring and prognosis assessment, and providing a basis for personalized medicine.

ctDNA positivity can serve as a strong predictor of decreased disease-free survival (DFS). Many studies in the CRC field (such as the GALAXY, BESPOKE, and DYNAMIC trials) have demonstrated that the level of ctDNA is closely related to the patient’s prognosis. Patients with persistently positive ctDNA or ctDNA conversion from negative to positive after surgery have a poor prognosis, while patients with ctDNA conversion from positive to negative have a significant survival benefits (Yu et al., 2025). The ctDNA mutation levels of 60 plasma samples were evaluated using NGS, and the results showed that patients with low ctDNA levels had a progression-free survival (PFS) of 12.3 months and an overall survival (OS) of 22.6 months, which were significantly longer than those of patients with high ctDNA levels (PFS = 5.8 months, OS = 9.4 months), suggesting that ctDNA can serve as a predictor of disease burden and treatment response (González-Medina et al., 2024).

The variant allele frequency (VAF) can reflect the degree of tumor clonal advantage. Hwang S et al. confirmed that the median OS of patients with VAF>3.9% in the NGS results of blood ctDNA was only 4.9 months, which was significantly lower than that of patients with VAF≤0.9% (16.4 months, p = 3.8 × 10^−7^) (Hwang et al., 2024). Allele frequency variance (AFV), as a measure of the uniformity of VAF distribution, can reflect the genomic instability (GI) of tumors. High AFV values are usually associated with greater tumor clonal heterogeneity, high chromosomal instability, and subclonal evolution, features that usually predict a poor prognosis. Liu R et al. proposed that AFV can be used as a new method to evaluate the prognosis of BTC. Patients with higher preoperative AFV levels had poorer OS (p = 0.004), and preoperative AFV showed better predictive value compared with postoperative AFV (Liu et al., 2024).

NGS is a commonly used ctNDA detection method in clinical practice, which can detect low-frequency mutations below 0.1%. At the same time, different panels of NGS can analyze the full spectrum of mutations such as point mutations, insertions and deletions (Indel), copy number variations (CNV), and fusion genes, reducing technical costs. Currently, NGS is widely used in MRD monitoring, prognosis assessment, and drug resistance mechanism research of CCA. However, recent studies have also showed that liver function status may affect NGS plasma ctDNA concentration determination (González-Medina et al., 2024). Studies have found that the NGS plasma ctDNA level in patients with liver dysfunction is 2.1 times higher than that in patients with normal liver function, which may be due to decreased liver metabolic capacity, resulting in reduced ctDNA clearance. Therefore, future studies should consider the impact of liver function on ctDNA load to avoid misjudging disease progression due to liver dysfunction.

### 4.3 Dynamic monitoring of CCA treatment

#### 4.3.1 Post-operative monitoring

Although radical resection can remove most of the tumor tissue, MRD may still exist, which cannot be detected by conventional imaging examinations. As a sensitive prognostic marker, ctDNA can effectively detect MRD and thus assess the risk of relapse in patients. Postoperative ctDNA detection can be used for early monitoring of CCA recurrence and provide a basis for adjuvant therapy decisions.

Compared with traditional markers and imaging examinations, ctDNA has higher sensitivity in predicting recurrence. In a study of 56 patients with curatively resected stage I-III BTC, blood ctDNA abnormalities were detected in 93.8% of recurrent patients at an average of 3.7 months in advance, and ctDNA had a stronger predictive ability for recurrence than traditional CA19-9 levels (HR = 1.17 [95% CI, 0.24 to 5.71]; P = 0.844) (Yu et al., 2025). A study by Reinert T et al. showed that ctDNA can detect recurrence several months earlier (174–222 days) than imaging examinations in monitoring tumor recurrence (Reinert et al., 2019).

A CCA-based subanalysis of the STAMP Phase II trial showed that ctDNA levels were positively correlated with tumor burden. During postoperative MRD monitoring, plasma ctDNA was evaluated by 16-plex PCR next-generation sequencing, and ctDNA positivity indicated that residual lesions were not completely eliminated and was significantly associated with worse DFS (HR = 1.8; 95% CI 1.06–3.07; p = 0.029) (Yoo Changhoon et al., 2024). During adjuvant chemotherapy, persistently positive ctDNA indicates that the patient will relapse 100% after surgery and their RFS is significantly shortened (Yoo C. et al., 2024).

These results show that ctDNA has a higher sensitivity in predicting recurrence than traditional biomarkers and imaging examinations. With the advancement of larger-scale prospective studies, tumor-specific ctDNA detection is expected to become an important recurrence monitoring biomarker in clinical practice and promote the establishment of a precise postoperative monitoring system.

#### 4.3.2 Chemotherapy and targeted therapy

Most CCA patients are already in the metastatic or locally advanced stage at the time of diagnosis, and chemotherapy (such as gemcitabine/cisplatin regimen) has become the standard treatment for unresectable or recurrent CCA. ctDNA plays an important role in this process. By monitoring the changes in specific gene mutations in ctDNA and the fluctuations in tumor load, it can reflect the patient’s response to treatment in real time. This can help patients optimize follow-up treatment strategies and provide timely personalized and precise treatment. Specifically, a decrease in ctDNA levels after chemotherapy indicates a reduction in tumor burden, indicating that the treatment is effective. If ctDNA turns negative after chemotherapy, it may indicate complete remission (CR) and the patient has a better treatment response. However, patients whose ctDNA levels do not decrease or continue to increase may indicate chemotherapy resistance or disease progression, with a higher risk of recurrence and significantly lower DFS than those whose ctDNA levels decrease. In addition, the study by Hwang S et al. showed that high variant allele frequency in ctDNA was closely associated with poor prognosis after gemcitabine/cisplatin chemotherapy, specifically manifested as a significant shortening of OS and PFS (p = 6.9 × 10^−6^ and p = 3.8 × 10^−7^) (Hwang et al., 2024). This study further demonstrated the important role of ctDNA in evaluating chemotherapy effects and prognosis.

Molecular targeted therapy and immunotherapy have gradually become the core of personalized treatment for CCA, especially in patients with specific gene mutations (such as FGFR2, IDH1, MSI-H, TMB-H) or ERBB2 gene amplification (Hwang et al., 2024). Currently, targeted therapy is widely used in patients with CCA, and dynamic monitoring of ctDNA can help predict the effect of targeted therapy. If the targeted mutation in ctDNA disappears or decreases, it usually means that the targeted therapy is effective and the patient is sensitive to the drug; if the targeted mutation does not decrease, it may indicate the presence of primary drug resistance, and the targeted therapy is not effective. However, patients may develop acquired resistance mutations during treatment, and ctDNA can be used to monitor the emergence of these resistance mutations, thereby helping to dynamically adjust treatment options (Goyal et al., 2017). Plasma NGS analysis results showed that approximately 34.3% of patients carried mutations that could be targeted for treatment, such as FGFR2, IDH1, MSI-H, and ERBB2, which makes ctDNA play an important role in personalized treatment decisions (Hwang et al., 2024). Individualized precision treatment is particularly critical for patients with different subtypes (Chen et al., 2024).

Currently, FGFR2 fusion mutations (approximately 10%–20%) and IDH1 gene mutations (approximately 15%–20%) are the most common mutations in CCA and have been widely used in clinical practice with FDA-approved targeted therapies (Abou-Alfa et al., 2020a; Uson Junior and Borad, 2022). Various FGFR inhibitors (such as infigratinib (Abou-Alfa et al., 2020a), Pemigatinib (Abou-Alfa et al., 2020b), Futibatinib (Goyal et al., 2023), and the recently observed Tasurgratinib (Morizane et al., 2025), etc.) have shown good therapeutic effects in CCA patients. However, patients with FGFR2 fusion may develop mutations such as FGFR2 V564F and N550K after receiving FGFR inhibitors, leading to drug resistance. Traditional ctDNA detection methods have a low sensitivity for FGFR2 fusion mutations, at only 18% (Vaidyanathan et al., 2018). However, there have been advances in ctDNA profiling techniques in recent years. Hwang S et al.'s study showed that ctDNA profiling can efficiently detect FGFR2 fusion mutations and comprehensively capture tumor genetic changes (Hwang et al., 2024). The optimized ctDNA method specifically targeting FGFR2 fusions (FGFR-Dx) developed by Julie W et al. has for the first time verified the feasibility of FGFR2 fusion detection at low VAF (0.5%) levels in BTC patients (Reeser et al., 2024). This method significantly improves the sensitivity of FGFR2 detection (92.9%, compared with the original 18%), and is lower in cost. It is expected to help more patients adjust their treatment plans in time when drug-resistant mutations appear early. The results of a randomized, double-blind, placebo-controlled phase 3 study (ClarIDHy) showed that ivosidenib can significantly improve the median PFS of patients with advanced IDH1 mutation CCA (2.7 months vs. 1.4 months, P < 0.0001), and has a good benefit in OS, but its objective response rate (tumor size reduction >30%) is only 2% (Abou-Alfa et al., 2020a). Patients receiving ivosidenib may develop new IDH1 R132C mutations or activating IDH2 mutations (R172V), resulting in drug failure (Harding et al., 2018; Lowery et al., 2019). ctDNA testing can identify these mutations in a timely manner, providing evidence for treatment adjustments.

#### 4.3.3 ctDNA monitoring of drug resistance

Drug resistance is one of the main reasons for CCA treatment failure. Drug resistance can be divided into primary resistance (no initial response to treatment) and acquired resistance (disease progression during treatment). Its potential mechanisms include secondary mutations of driver genes (such as EGFR, PI3K/Akt, Erk and NF-κB), epigenetic remodeling (such as abnormal DNA methylation) and cell apoptosis escape (Varghese et al., 2021). These resistance mechanisms not only affect the efficacy of chemotherapy and targeted therapy, but may also accelerate tumor metastasis and recurrence. Therefore, real-time tracking of drug resistance is crucial for developing individualized treatment plans and evaluating patient prognosis. ctDNA can break through the limitations of a single tissue biopsy and simultaneously monitor multiple drug resistance mechanisms, providing a basis for dynamically adjusting treatment plans. A study of 42 patients with advanced gastrointestinal tumors showed that among 23 resistant patients, the positive rate of detecting blood ctDNA drug-resistant mutations using NGS and ddPCR reached 87% (20/23), significantly higher than the 48% (11/23) of tissue biopsy. The proportion of ctDNA detection of multiple drug resistance mechanisms (40%) was 4.4 times higher than that of tissue biopsy (9%) (Parikh et al., 2019). Nevertheless, the clinical significance of ctDNA in monitoring drug resistance in CCA has not yet been fully confirmed, and more prospective studies are still needed to verify its application value.

### 4.4 CCA metastasis detection

CCA is highly invasive and metastatic, with common metastatic sites including the liver, peritoneum, lymph nodes, and lungs. Metastatic disease is usually associated with progressive disease and a poor prognosis. Therefore, early detection of metastatic lesions is crucial to improve the prognosis of CAA. Traditional imaging methods (such as CT and MRI) are difficult to detect small and occult metastatic lesions in time. The level of ctDNA is closely related to the degree of tumor differentiation. Poorly differentiated tumors are usually more metastatic. However, it has no significant correlation with the number of metastatic sites (Pietrasz et al., 2017). The concordance between ctDNA and metastatic tissue was significantly higher than that between primary tumors (Kruger et al., 2018). In patients with CCA, distant metastases such as the liver, lung, and peritoneum are often accompanied by increased plasma ctDNA abundance. Different metastatic sites are often accompanied by different types of ctDNA mutations: TP53 mutations are more common in intrahepatic metastasis; KRAS mutations are common in lung metastasis, indicating enhanced tumor invasiveness; peritoneal dissemination is associated with inactivation mutations of CTNNB1 and BAP1, suggesting that drug resistance may increase (Amato et al., 2019). Furthermore, ctDNA metastasis did not differ significantly with patient gender, age or metastatic site, further enhancing its broad applicability as a metastasis monitoring tool (Del Re et al., 2017).

## 5 Challenges and limitations of ctDNA testing

As an emerging liquid biopsy technology, ctDNA detection has shown great potential in early diagnosis of tumors, efficacy evaluation, and recurrence monitoring. However, it still faces many challenges and limitations in practical applications, mainly including the following aspects.

### 5.1 Technical sensitivity and specificity issues

The abundance of ctDNA in the blood is low in the early stages of tumors or in the remission stage after treatment, and may be as low as 0.1% (Crowley et al., 2013; Anagnostou and Velculescu, 2024; Zhang et al., 2024). Targeted methods for detecting ctDNA have reached their current biological limits. Although existing detection technologies (such as dPCR, NGS, etc.) continue to improve, it is still difficult to achieve high-sensitivity detection in low-abundance ctDNA (Kim et al., 2023; Normanno et al., 2025). Clonal hematopoiesis (CH) is a condition in which somatic mutations (such as mutations in genes such as DNMT3A, TET2, and ASXL1) exist in healthy individuals. cfDNA produced by clonal hematopoietic cells may interfere with ctDNA detection, leading to false positives (Imai et al., 2022). Studies have shown that clonal hematopoietic (CH) mutations were detected in the plasma of 29.8% of patients with gastric cancer, 10% of prostate cancer, and 30% of patients with metastatic renal cell carcinoma, with a high risk of misdiagnosis (Bacon et al., 2020; Jensen et al., 2021; Lee K. S. et al., 2024). Currently, there is a lack of mature methods to exclude clonal hematopoietic (CH) variants. Inclusion of clonal hematopoietic (CH) variants in commercial ctDNA targeted panels is expected to help distinguish true ctDNA from other somatic expansions *in vivo* (Bacon et al., 2020).

### 5.2 Sample collection and standardization problems

Although blood-based ctDNA analysis shows great promise in molecular diagnosis of various cancer types, the ratio and stability of ctDNA are low, and various stages such as collection and analysis greatly affect the results (Lampignano et al., 2020; Pascual et al., 2022; Shin et al., 2022). Paul et al. divided the key parts of ctDNA analysis workflow into two parts: pre-analysis and analysis. The key parts of pre-analysis include: sample collection, storage, transportation, elution and extraction of ctDNA, etc.; the key parts of analysis workflow include ctDNA quantification, analysis input and identification (van der Leest and Schuuring, 2024). Although the current international guidelines (the “Use of Circulating Tumor DNA for Curative-Intent Solid Tumor Drug Development Guidance for Industry” issued by the FDA) have put forward framework recommendations, the ctDNA enrichment and detection schemes for different cancer types (such as lung cancer vs. bile duct cancer) still need to be refined. Establishing a full-process quality control system from sample collection to reporting will be the key to clinical promotion.

### 5.3 Technical cost and multi-center verification issues

The technology of ctDNA detection is complex and involves multiple high-investment processes. The high technical cost is one of the important reasons that limits its large-scale clinical application. Deep sequencing and personalized sequencing technologies are expensive, and body fluids such as pancreatic juice and bile require special extraction reagents, which also increase costs. A retrospective observational cohort study from Italy showed that tumor profiling using comprehensive NGS panels improved patients’ eligibility to personalized therapies (De Micheli et al., 2025). However, the diagnostic cost per patient ranges from $5500 to $8400 (NSCLC: $8400; CCA: $5500; PC: $6600).

Many different ctDNA tests claim to have specificity and sensitivity, but there are few independent or cross-platform validation studies. One report showed that a very poor ctDNA correlation was found between liquid biopsy platforms Guardant360 and PlasmaSELECT when examining 42 genes that overlapped between the two platforms (Torga and Pienta, 2018). Although this was a small study involving 40 metastatic prostate cancer patients, 25 of 40 had alterations in overlapping genes, only three had complete congruence, six had partial congruence, and sixteen had no congruence. Differences in detection methods, differences in instrument sensitivity, and lack of standardization in threshold setting make multi-center validation of ctDNA still challenging. Unfortunately, there is currently a lack of research in this area (Cherney et al., 2025).

### 5.4 Tumor heterogeneity and dynamic changes

Different stages and anatomical locations of CCA may lead to differences in genetic information between ctDNA, and this heterogeneity increases the complexity of ctDNA detection. In AJCC stage II and distal CCA, the sensitivity of ctDNA in detecting copy number variations (CNVs) is significantly reduced (Shen et al., 2019). The concordance between ctDNA and tissue results was high, 74% overall, and 92% for iCCA, but was lower for eCCA (55%) (Ettrich et al., 2019). Oliver et al.'s study showed that the diagnostic accuracy and sensitivity of ctDNA detection in advanced CCA were as high as 97.7% and 92.3%, and the specificity was 100%. However, relevant clinical research is still needed in patients with early CCA (Zill et al., 2015). In conclusion, tumor heterogeneity, along with variations in CCA stages and types, compromises ctDNA detection performance, thereby restricting its clinical applicability.

ctDNA levels are affected by multiple factors such as tumor burden and therapeutic intervention, resulting in significant dynamic changes. In a small sample clinical study conducted by Kyung et al., ctDNA samples from 16 patients with CCA were analyzed after surgery, and new mutations were found in ctDNA of eight patients (50%) after surgery (Kim et al., 2023). Postoperative plasma mutations had a sensitivity of 44% and a specificity of 45% for detecting clinical relapses. Ettrich et al. performed ctDNA testing in 11 patients who received first-line palliative chemotherapy. Compared with baseline, 36% (4/11) of these patients had a change in the ctDNA mutational landscape. Three of them had TP53 mutations, which were not detected after treatment. The fourth patient had a PBRM1 mutation, which no longer appeared at the “progression” time point (Ettrich et al., 2019). Both studies demonstrated the potential of ctDNA testing in monitoring CCA treatment response and elucidating drug resistance mechanisms; however, their clinical generalizability is limited by small sample sizes and a single-institution study design.

## 6 Future research directions

### 6.1 Multiplex detection of ctDNA combined with other liquid biopsy markers

#### 6.1.1 ctDNA-CA19-9 combined detection

In addition to traditional tumor markers, recent studies have also found that the level of syncytin-1 in bile cfDNA can be used as a new biomarker, especially with potential in the early diagnosis of CCA. The study by He JD et al. showed that syncytin-1 performed better than CA19-9, CEA and AFP in the diagnosis of bile cfDNA, with an AUC of 0.805 (95% CI: 0.719–0.890, p < 0.001), a specificity of 90.2% and a sensitivity of 75%. Combining syncytin-1 with CA19-9 increased the AUC to 0.927 (0.901 95% CI: 0.877–0.978, p < 0.001) (He et al., 2025). The results show that the combination of the two can significantly improve the efficiency and accuracy of early diagnosis, providing an important basis for the early diagnosis of CCA.

#### 6.1.2 ctDNA-cf-miRNA combined detection

In recent years, cell-free miRNA (cf-miRNA), like ctDNA, has shown promise in diagnosing a variety of diseases, including heart disease, infection, and various cancers (Biró et al., 2020; Song et al., 2020; Hadipour et al., 2023). Studies have shown that there is a significant positive correlation between ctDNA and cf-miRNA (Prasopdee et al., 2024). The combination of plasma cf-miRNA and ctDNA can significantly improve the sensitivity and specificity of differential diagnosis of CCA compared with ctDNA detection alone (83.33%, 100% vs. 75.00%, 95.83%). This means that the combination of the two can serve as a potential diagnostic tool to distinguish CCA from other diseases and further improve the accuracy of CCA diagnosis.

#### 6.1.3 ctDNA-CTC combined detection

Circulating tumor cells (CTCs) spread in the bloodstream through epithelial-mesenchymal transition and are closely related to tumor metastasis. They have been actively studied as prognostic biomarkers (Han et al., 2021). A study conducted by Sung et al. demonstrated that the combined detection of CTC and ctDNA improved the accuracy of predicting recurrence in BTC patients after standard treatment (surgery + adjuvant chemotherapy) (AUC = 0.92, sensitivity 93.8%, specificity 87.5%), which was much higher than CA 19–9 (AUC = 0.75, sensitivity 75.0%, specificity 75.0%) (Park et al., 2024).

The multiplex detection scheme of ctDNA combined with other liquid biopsy markers is still in its infancy, and the data integration standards of different detection platforms have not yet been unified. In the future, with the help of artificial intelligence (AI) algorithms and the development of multicenter cohorts, the clinical operability of combined markers can be further verified.

### 6.2 Application of ctDNA in immunotherapy

#### 6.2.1 Predicting response to immunotherapy

ctDNA detection can analyze the dynamics of tumor clonal evolution in real time, overcome the limitations of traditional imaging in evaluating immunotherapy response (such as pseudoprogression, delayed response, etc.), and provide a non-invasive means to monitor tumor dynamics and immunotherapy response (Weber et al., 2024).

Li et al. developed a patient-specific ctDNA fingerprint based on NGS (including eight hotspot genes: BRAF, EGFR, ERBB2, KIT, KRAS, MET, NRAS, and PIK3CA), by monitoring ctDNA content fraction (CCF) levels and CCF fold changes. This method has improved the specificity and sensitivity of monitoring the response to immunotherapy in CCA and can identify tumor recurrence earlier than imaging (Li et al., 2020).

Xu et al. constructed a CNV risk scoring model based on copy number variation (CNV) in plasma ctDNA to predict the clinical outcomes of immune checkpoint inhibitor (ICI) treatment in advanced hepatobiliary cancer. The results showed that the low CNV risk group had better clinical outcomes than the high CNV risk group (median PFS: 6.17 months vs. 2.60 months, HR = 0.045; median OS: not reached vs. 6.5 months, HR = 0.39) (Yang X. et al., 2021).

#### 6.2.2 Guidance on perioperative immunotherapy strategies

Yu et al. used ctDNA analysis to guide the adjuvant treatment of a patient with stage III CCA after resection and achieved good results. The patient started standard capecitabine adjuvant therapy on the 50th day after surgery. ctDNA was positive for two consecutive times and CA19-9 continued to rise. Before obtaining evidence of radiological recurrence, the patient was given capecitabine combined with pembrolizumab. After 112 days, ctDNA was negative and CA19-9 returned to normal after 155 days (Yu et al., 2024). This case report reveals the guiding significance of ctDNA testing for perioperative adjuvant treatment of CCA, but due to the limited sample size, the results are less credible.

Currently, there are few studies on ctDNA and CCA immunotherapy. In the future, larger-scale prospective studies are needed to demonstrate the role and clinical application value of ctDNA in CCA immunotherapy.

## 7 Conclusion

As an emerging tumor liquid biopsy technology, ctDNA detection has broad application prospects in CCA. ctDNA has the advantages of high tissue consistency and high sensitivity, and has shown its effectiveness in early screening and diagnosis. In the future, the combined application of ctDNA and other tumor liquid biopsy markers is expected to achieve more efficient and accurate early diagnosis of CCA. Moreover, due to the easy availability and short half-life of ctDNA samples, real-time treatment response monitoring and prognosis assessment can be provided for patients receiving treatment. In terms of treatment, by monitoring the levels of various targeted mutations such as FGFR2 and IDH1 in patients, drug resistance can be detected in a timely manner, providing patients with personalized treatment plans. In the future, there is still an urgent need to improve the ctDNA detection standards and standardize each link of sample collection, analysis and detection. As a ray of hope in CCA treatment, immunotherapy is increasingly widely used in clinical practice. ctDNA testing is expected to further broaden the use scenarios of immunotherapy, but currently there are few studies in this field and the sample size is small. In the future, there is an urgent need to conduct more clinical studies with larger sample sizes.

## Glossary

ctDNA: Circulating tumor DNA
CCA: Cholangiocarcinoma
iCCA: intrahepatic cholangiocarcinoma
eCCA: extrahepatic cholangiocarcinoma
dCCA: distal cholangiocarcinoma
OS: Overall survival
cfDNA: Cell free DNA
rt-qPCR: real-time quantitative PCR
ddPCR: digital droplet PCR
NGS: Next-generation sequencing
TAm-Seq: Tagged amplicon deep sequencing
CAPP-Seq: Cancer personalized profiling by deep sequencing
WGS: Whole genome sequencing
WES: Whole exome sequencing
WGBS: Whole genome bisulfite sequencing
MRD: Minimal residual disease
PFS: Progression-free survival
TME: Tumor microenvironment
BTC: Biliary tract cancer
EUS: Endoscopic ultrasound
MRCP: Magnetic resonance cholangiopancreatography
ERCP: Endoscopic retrograde cholangiopancreatography
CA19-9: Carbohydrate antigen 19–9
DF: Duodenal fluid
PPV: Positive predictive value
MS-HRM: Methylation-sensitive high-resolution melting
RRBS: Reduced-representation bisulfite sequencing
ICI: Immune checkpoint inhibitor
CEA: Carcinoembryonic antigen
MRD: Molecular residual disease
DFS: Disease-free survival
AFV: Allele frequency variance
RFS: Recurrence-free survival
MAF: Mutant allele frequency
HR: Homologous recombination repair
CH: Clonal hematopoiesis
CNVs: Copy number variations
CTCs: Circulating tumor cells
CCF: ctDNA content fraction
NSCLC: Non-small Cell Lung Cancer
PC: Pancreatic Cancer
